# Dissecting the mechanisms of CASK deficiency on the clearance of Aβ in microglia

**DOI:** 10.1002/alz.086751

**Published:** 2025-01-03

**Authors:** Jacky Chang, Yen Ting Cho, Yu Yi Lin, Irene Han‐Juo Cheng

**Affiliations:** ^1^ Institute of Brain Sciene, National Yang Ming Chiao Tung University, Taipei Taiwan

## Abstract

**Background:**

Amyloid‐beta (Aβ) deposition is a key pathological characteristic of Alzheimer’s disease (AD). Microglia serves as a crucial system responsible for clearing Aβ. Activated microglia migrate towards Aβ deposits, engulf them, and breakdown Aβ through cathepsins within the lysosome. Calcium/calmodulin‐dependent serine protein kinase (CASK) regulates microglia’s migration and phagocytic activity. Notably, our findings revealed a reduction in the expression of microglial CASK upon Aβ treatment. Nevertheless, the specific function of CASK in activated microglia remains undiscovered.

**Method:**

The chemotaxis and migration of CASK KO BV2 microglia were examined via transwell and wound healing assays with Aβ presence variations. Focal adhesion kinase (FAK) protein levels were assessed through western blotting. Phagocytic capacity will be tested by using fluorescence‐labeled Aβ and microbeads after activated by LPS and Aβ. Vinculin, CD68 or lysosome associated membrane (LAMP1) were visualized using immunostaining. Actin was stained with phalloidin. The colocalization was measured by ImageJ. Expression of cathepsin B, D, and S, and Aβ‐degrading enzymes in CASK KO microglia were measured via qPCR. Lysosome morphology was tracked using lysotracker.

**Result:**

The expression of microglial CASK was reduced upon Aβ treatment. The chemotaxis and migration capabilities of CASK KO microglia are notably inferior to WT microglia. After Aβ treatment, FAK protein levels were significantly higher in WT but not CASK KO microglia. Moreover, CASK KO microglia had lower vinculin‐lamellipodia colocalization and lamellipodia area than WT microglia. Conversely, CASK deficiency in microglia increased Aβ engulfment and the number of lysosomes. In addition, LAMP1‐Aβ colocalization was diminished in CASK KO microglia. CASK KO microglia had higher levels of cathepsin B, D, and S, as well as and Aβ‐degrading enzymes than WT microglia.

**Conclusion:**

Our investigation revealed the critical roles of CASK in regulating microglial migration and phagocytic functions, particularly in the context of Aβ treatment. These findings necessitate further exploration into the nuanced functions of CASK in activated microglia, potentially offering insights into novel therapeutic avenues for AD.

